# Rapid Elemental Analysis and Provenance Study of *Blumea balsamifera* DC Using Laser-Induced Breakdown Spectroscopy

**DOI:** 10.3390/s150100642

**Published:** 2014-12-31

**Authors:** Xiaona Liu, Qiao Zhang, Zhisheng Wu, Xinyuan Shi, Na Zhao, Yanjiang Qiao

**Affiliations:** Beijing University of Chinese Medicine, South of Wangjing Middle Ring Road, Chaoyang District, Beijing 100102, China; E-Mails: xiaonaliu5627@163.com (X.L.); zhangqiao0824@foxmail.com (Q.Z.); shixinyuan01@163.com (X.S.); zn26140@163.com (N.Z.)

**Keywords:** LIBS, *Blumea balsamifera* DC, provenance study, PCA, PLS-DA

## Abstract

Laser-induced breakdown spectroscopy (LIBS) was applied to perform a rapid elemental analysis and provenance study of *Blumea balsamifera* DC. Principal component analysis (PCA) and partial least squares discriminant analysis (PLS-DA) were implemented to exploit the multivariate nature of the LIBS data. Scores and loadings of computed principal components visually illustrated the differing spectral data. The PLS-DA algorithm showed good classification performance. The PLS-DA model using complete spectra as input variables had similar discrimination performance to using selected spectral lines as input variables. The down-selection of spectral lines was specifically focused on the major elements of *B. balsamifera* samples. Results indicated that LIBS could be used to rapidly analyze elements and to perform provenance study of *B. balsamifera*.

## 1. Introduction

*Blumea balsamifera* DC, a medicinal herb, is widely distributed in Eastern and Southeastern Asia. It has been used in folk medicine by Chinese minority groups for the treatment of septic wounds, respiratory infections, and stomach pains [1–3]. This herb is also useful for the treatment of fever, lumbago, increasing appetite, skin diseases, wounds, liver cirrhosis, and kidney stones [4]. *B. balsamifera* is the main plant source of natural borneol. The main provenances of *B. balsamifera* are Hainan and Guizhou provinces in China. Provenance study of herbal medicinal species is usually restricted to the detection of a few active components using high-performance liquid chromatography (HPLC) and gas chromatography-mass spectrometry (GC-MS), *etc.* Compared to the current fingerprint studies of organic compositions, fewer studies on inorganic elements have been reported. However, the quality and efficacy of herbal medicinal species are somewhat uneven due to disparities among conditions, such as soil and climate in different cultivation areas [5]. The elemental composition of a plant has a great impact on its quality, and depends on its provenance, therefore, a rapid and accurate elemental analysis method is necessary to study the provenance of medicinal herbs.

Laser-induced breakdown spectroscopy (LIBS) is a versatile spectroscopic technique [6,7] that uses the light emitted from laser-induced microplasma to determine the elemental composition of samples. A high-power laser beam is focused on the surface of the sample. A small amount of the sample material is ablated and a luminous laser-induced plasma (LIP) is created. The LIP consists of atoms and ions from the ablated material. Molecules are created in the plasma during cooling. Spectral characteristics allow quantitative analysis under well specified conditions. Compared to other traditional techniques, *i.e.*, flame atomic absorption spectrometry (FAAS), laser ablation inductively coupled plasma mass spectrometry (ICP-MS), and inductively coupled plasma atomic emission spectrometry (ICP-OES), LIBS has unique advantages, such as requiring no or little sample preparation, *in situ* and even remote element detection. These characteristics are particularly valuable in the analysis of chemical and hazardous samples [8,9]. Moreover, the significant capability of LIBS for material characteristics is chemical imaging with a high spatial resolution [10]. Recently, LIBS has emerged as a powerful technique for direct multi-element analysis of various samples in solid, liquid, and gaseous phase. Several books [11,12] and reviews [6,7,13,14] have been published about LIBS, ranging from fundamental theory to applied research. LIP spectra are characterized by a tremendous amount of spectral lines. Such spectra give information about the complex elemental composition of the sample, *i.e.*, a “chemical fingerprint”. Thus, strong requirements are imposed on the efficient and effective handling a large amount of data.

Multivariate analysis is able to reduce multi-dimensional data to lower dimension factors that describe the variance among samples. It is useful in identification, classification and quantitative analysis. For example, partial least squares discriminant analysis (PLS-DA), soft independent modeling of class analogy (SIMCA) and support vector machines (SVM) were all used to classify pharmaceutical samples [15]. Linear and rank correlation methods were employed to discriminate glass samples for forensic applications by LIBS [16]. In particular, PLS-DA is a more viable chemometric analysis for discrimination of heterogeneous residue samples with similar composition. Ollila *et al.* [17] compared two PLS-DA algorithms for identifying geological samples using the ChemCam LIBS instrument. Xia *et al.* [18] investigated the potential of the technique for classification of waste materials in concrete recycling and found that PLS-DA achieved full discrimination. Schröder *et al.* [19] demonstrated that PLS-DA was a suitable analytical technique for the investigation of salts and frozen salt solutions by LIBS under martian atmospheric conditions. Based on the elemental “fingerprint” and multivariate analysis, LIBS technique was able to discriminate geological materials [20]. Akpovo *et al.* [21] used principal components analysis (PCA), discriminant function analysis (DFA), and hierarchical cluster analysis (HCA) to discriminate the geographical origin of oysters. Alvey *et al.* [22] applied a PLS-DA algorithm for classification of garnet samples based on geochemical fingerprinting via LIBS.

Numerous studies of the LIBS technique have been performed in plants [23–25]. Trevizan *et al.* investigated the micro- and macronutrients of plant materials by means of LIBS [26,27]. Krystofova *et al.* [28] reported the distribution of lead in sunflower plants using the LIBS technique. Galiová *et al.* [29] demonstrated the feasibility of mapping the distribution of silver and copper in plant samples (leaves of *Helianthus annuus* L.) by LIBS. Additionally, molecular bands can be used for analysis of biomaterials in LIBS experiments. Singh *et al.* [30] reported the prospects for using LIBS in biomedical applications. However, the application of LIBS for analysis of medicinal herbs has not been well studied yet [13,31]. Moreover, there is an increasing interest in the use of medical herbs throughout the Western Hemisphere. More than 500 kinds of Chinese herbs are found in the Chinese Pharmacopoeia [32]. Provenance or geoherbalism is a critical determination of quality in medical herbs.

This study investigated the feasibility of LIBS for rapid analysis of medical herbs composition and their provenance. Specifically, the aim of this work was to improve the recognition capacity for classifying similar *B. balsamifera* samples. PCA was employed to assess the data structure in the multivariate space. PLS-DA was selected because it can significantly improve the identification capability without considerably increasing the difficulty of implementation. The variables contributing to the classification in the PLS-DA model was explored through examining the variable importance in projection (VIP) scores.

## 2. Materials and Methods

### 2.1. Materials

Dried aerial parts including leaves and stems of *B. balsamifera* (Ai-na-xiang) samples were provided by the Tropical Crops Genetic Resources Institute (Chinese Academy of Tropical Agricultural Sciences, Beijing, China), as shown in Table 1. These materials were mainly collected from two geographical regions in P.R. China and were identified by Dr. Fu-Lai Yu (Tropical Crops Genetic Resources Institute, Chinese Academy of Tropical Agricultural Sciences). A voucher specimen (No. GT022) was kept at the Key Laboratory of TCM-Information Engineering of the State Administration of TCM, Beijing University of Chinese Medicine.

The homogeneity of samples is critical to the reproducibility of LIBS analysis. Grinding was used to reduce particle size and ensure the homogeneity of the sample surface for micro-analysis [33]. Before LIBS measurement, samples were finely ground (typical grain size, <150 μm). The ground powder was pressed into pellets (13 mm diameter and 4 mm thick) using a hydraulic pellet press with 10 tons of pressure.

### 2.2. Equipment and Parameter Conditions

**Figure 1.**
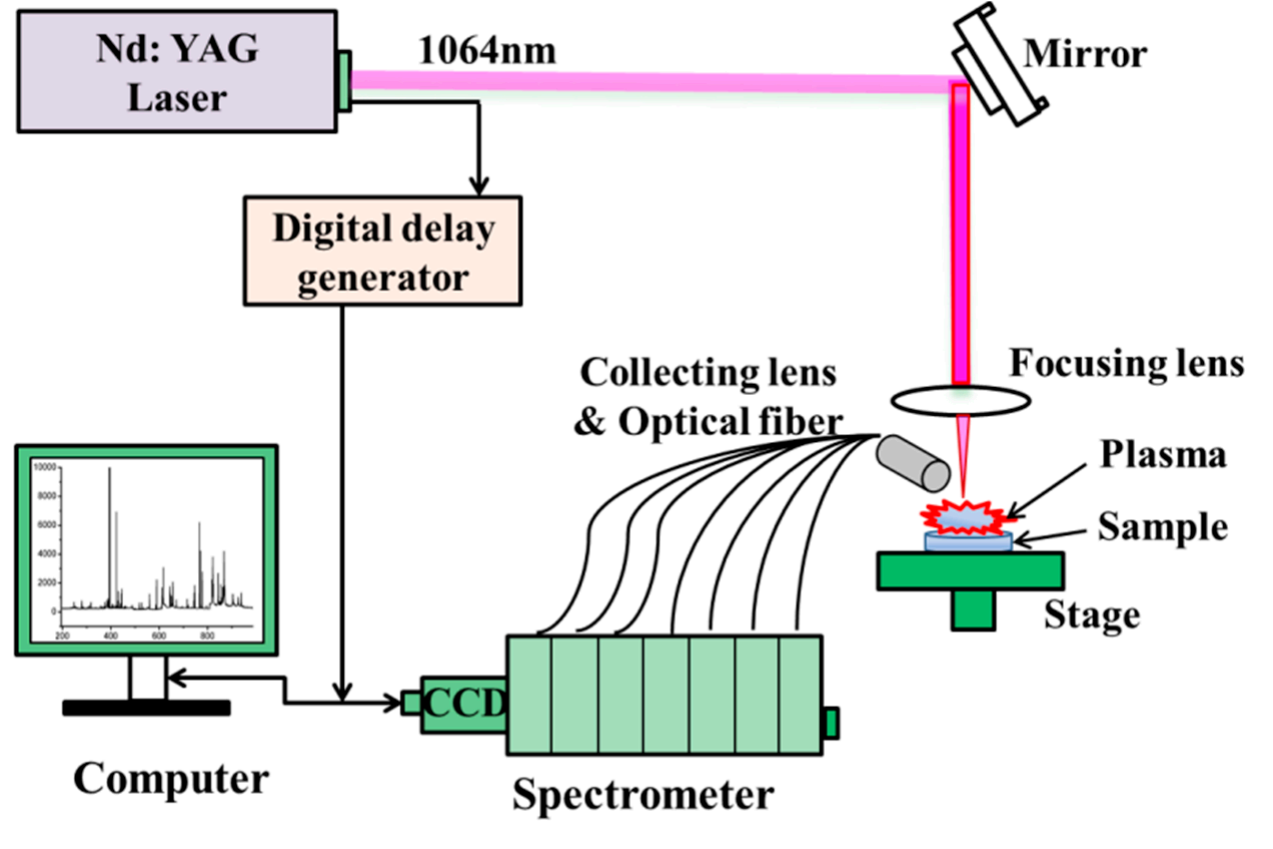
Schematic diagram of LIBS system.

A commercially available LIBS instrument with a spectrometer (ChemReveal™-3764, TSI Inc., St Paul, MN, USA) was employed to detect a total of 95 *B. balsamifera* samples. The experimental setup is schematically shown in Figure 1. A Q-switched Nd:YAG laser (New Wave Research, Fremont, CA, USA) with a laser power of 90 mJ/pulse and 1–3 ns pulse duration (FWHM) at 1064 nm was used in the experiment. The spectral range of this system was ∼167–984 nm with an approximate resolution of 0.1 nm. The laser beam passed through a pierced parabolic mirror and was focused vertically onto the sample. The sample surface was expanded by a telescope (LMH-5X-1064, Thorlabs Inc., Newton, NJ, USA). The size of laser spot was about 100 μm diameter. Plasma light was collected by a 50 mm focal length lens. The radiation was then introduced on the entrance slit of a spectrometer (3764 XRF, Scientific Ltd., Osborne Park, WA, Australia) via a small lens and optical fibers. The emission spectrum was recorded with a high-resolution seven channel-charge-coupled device (CCD) camera. A *x-y-z* translation stage was used to ensure movement of the sample to a fresh spot. Experiments were performed in ambient air with a gate width of 1 ms and a detector delay of 1 μs.

### 2.3. Data Acquisition

LIPs were produced on the surface of *B. balsamifera* samples. Radiation of luminous LIP was collected, spectrally resolved and used for further spectroscopic analysis. For each sample, five positions were randomly selected and each position consisted of nine laser pulses (3 × 3 matrix∼100 μm × 100 μm). As a result, a total of 45 LIBS spectra per sample were acquired. To minimize the influence from sample heterogeneity and other fluctuation factors, in our measurements the measured spectra were averaged per sample.

### 2.4. Multivariate Analysis

It was chosen to do no further data pretreatment other than mean-centering [17,34]. Since the underlying background structure contains inherent features of spectra and should therefore be useful for discrimination and identification of different samples. PCA and PLS-DA were performed by PLS_toolbox version 6.21 under Matlab version R2009a (MathWorks Inc., Natick, MA, USA).

PCA is an unsupervised technique that can be used in the first instance to detect outliers of different classes [35,36]. It is commonly utilized for data exploration to reveal hidden patterns and major trends. Similarities and differences can be found and variables responsible for these are unveiled. PCA transforms variables (the data in matrix X), such as the intensity into linear combinations called principal components (PC) that describe the variance within the data. The coordinates among the independent PC scores can help to visualize similarities of spectra. If intra-class variance equals or exceeds inter-class variance then classification performance of PCA becomes suboptimal.

PLS-DA is a supervised classification algorithm [18,21]. The input variables were complete spectra and selected spectral lines. Pre-treated datasets were then divided into training and test sets by using the classic Kennard-Stone (KS) algorithm [37]. Leave-one-out (LOO) cross-validation paradigm was applied. PLS-DA decomposes the spectra as linear combinations of components, and the scores are correlated to both intra-class variance and inter-class variance [21,38,39]. Two input matrices are used to construct the PLS-DA model. The X block contains input matrix (complete spectra or selected spectral lines) for modeling. The Y block contains the class information of each spectrum (positive for one class, negative for the other class). The output of the PLS-DA models is the Y predictor block, which estimates the probability of class affiliation. In this case, one class was Hainan and the other class was Guizhou. To obtain good discrimination while avoiding model overfitting, the number of LVs was determined by 10-fold cross-validation. The optimal number of latent variables (LVs) was selected based on the first minimum classification error [22,26]. Subsequently, the test set was used to evaluation performance. The spectra were assigned to the class with the highest probability.

### 2.5. Evaluation of Classification Performance

Sensitivity and specificity are two basic parameters for measuring the accuracy of a diagnostic test [34,38]. Sensitivity is called the true positive rate (TPR). Specificity is defined as the proportion of correctly identified, fault-free recognitions. The values of both metrics (sensitivity and specificity) are indispensable in evaluating a screening process, as neither one can properly evaluate the process alone. Accuracy is another main parameter of a recognition procedure, expressed as the fraction of correctly classified samples in the total amount of samples [35]. More detailed description of these metrics is provided in [35,40]. Sensitivity, specificity, and total accuracy of a detection system are calculated according to Equations (1)–(3)

(1) sensitivity=TP/(TP+FN)

(2) specificity=TN/(TN+FP)

(3) Total accuracy=(TN+TP)/(TP+FN+TN+FP)

where TP is true positive classification, FP is false positive classification, TN is true negative classification and FN is false negative classification.

## 3. Results and Discussion

### 3.1. LIBS Spectra

**Figure 2.**
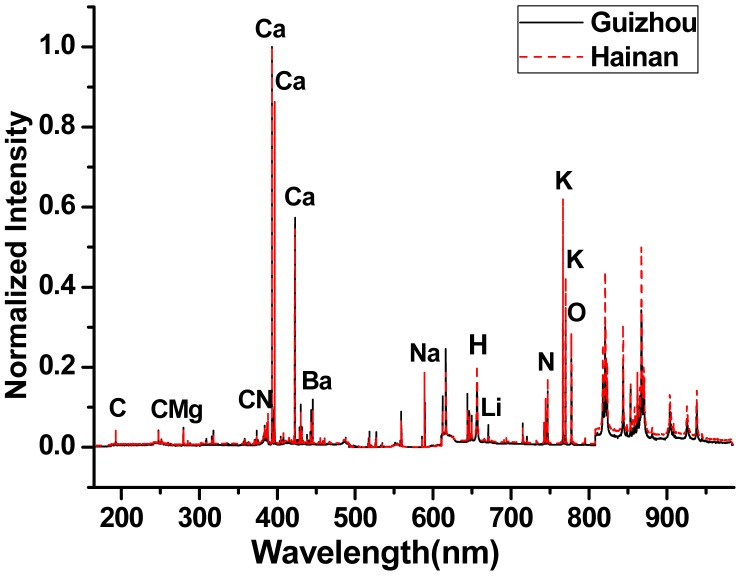
Normalized LIBS spectra of the *B. balsamifera* samples from Hainan and Guizhou provinces.

Figure 2 shows the LIBS average spectra of *B. balsamifera* from Hainan and Guizhou provinces. The identification of the atomic, ionic transitions and molecular bands were performed by using the NIST atomic lines database and relative references. The 48 selected spectral lines including 13 elements and two molecular bands are shown in Table 2. In the spectral region, atomic and ionic emission lines of the major mineral elements (Ca, K, Na and Mg) as well as Al were observed. All spectra showed strong emission lines from Calcium (Ca(II) 393.375 nm, Ca(II) 396.816 nm and Ca(I) 445.441 nm), sodium (Na(I) 588.952 nm), hydrogen (H(I) 656.315 nm), potassium (K(I) 766.523 nm, K(I) 769.959 nm), to nitrogen (N(I) 746.918 nm) and oxygen (O(I) 777.212 nm, O(I) 777.492 nm).

The molecular emission bands of CN violet bands were indicated in Figure 2. As many of the same emission lines were observed, it was difficult to visually discriminate differences between the spectra.

### 3.2. PCA Model

**Figure 3.**
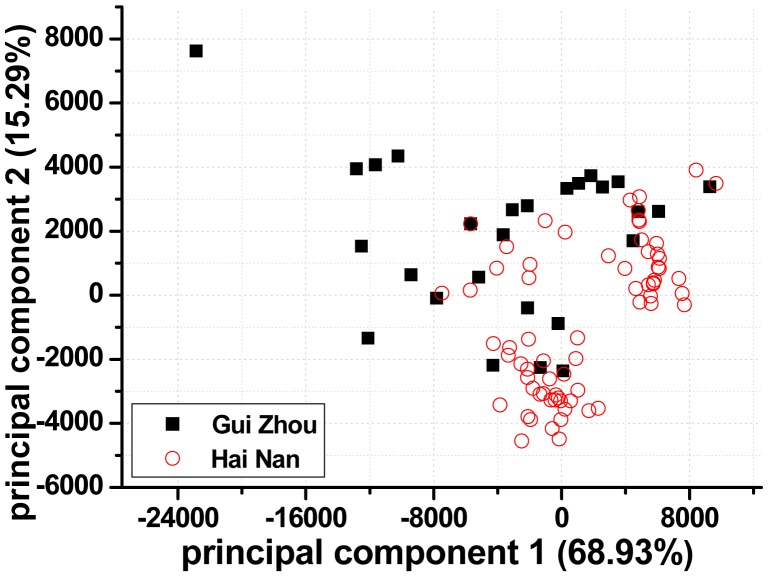
Principal components analysis of spectra from the 95 samples (projection on the first two principal components).

PCA provides a visual representation of relationships between samples and samples as well as between samples and variables. For visual clarity, the PCs were calculated using complete spectra (mean-centering pretreatment). The output of PCA resulted in three uncorrelated and orthogonal PCs that accounted for 90.76% of the total variability among samples. Figure 3 shows the PCA scores along the first two principal components (PC), which represented 84.22% of the total variability. The dispersion and local overlapping groups of data points in the PC score plots may be ascribed to the large chemical and mineralogical variability within each provenance. Outliers are detectable since they are relatively far from other spectra. Clearly, one spectrum lied apart from other spectra of its class (as shown in Figure 3). Consequently, the spectrum was eliminated from the Guizhou training set.

### 3.3. PLS-DA Model

**Figure 4.**
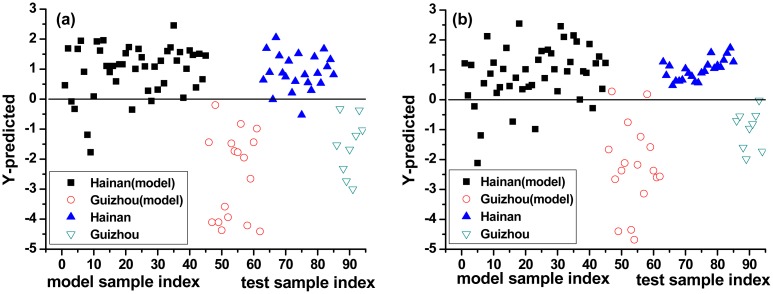
(Color online) Graphical representations of the cross-validation PLS-DA performed on datasets. (**a**) PLS-DA model 1# (complete spectra); and (**b**) PLS-DA model 2# (selected spectral lines).

PLS-DA considers the VIP scores for discrimination of LIBS spectra. One PLS-DA model was constructed using complete spectra (mean-centering pretreatment) and was called PLS-DA model 1#. A second PLS-DA model was constructed using selected spectral lines and was called PLS-DA model 2#. The previously identified outlier was removed from the calibration set, which then had 94 spectra. The results of two PLS-DA models are shown in Figure 4.

The y-axis represents the class prediction (Hainan or Guizhou) and the x-axis is simply the sample index. A detection threshold (0) was established by the model based on the 94 samples. Figure 4 illustrates the probability that a sample belongs to Hainan or Guizhou class for PLS-DA model 1# (Figure 4a) as well PLS-DA model 2# (Figure 4b).

**Figure 5.**
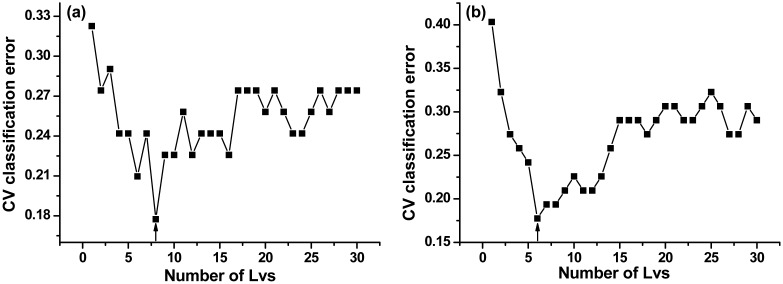
The plots of cross-validation classification error *versus* the number of LVs. (**a**) PLS-DA model 1# (complete spectra); and (**b**) PLS-DA model 2# (selected spectral lines).

The optimal numbers of LVs were 8 and 6 in the two PLS-DA models, respectively (Figure 5a, b).

For the training set, PLS-DA model 1# showed similar performance to PLS-DA model 2#. Specificity, sensitivity and total accuracy were 100%, 86.67%, and 90.32% for PLS-DA model 1#, and were 86.67%, 88.24%, and 87.10% for PLS-DA model 2#, respectively. For the test set, PLS-DA model 2# had slightly better performance than PLS-DA model 1#. Specificity, sensitivity, and total accuracy were 100%, 91.30%, and 93.75% in PLS-DA model 1#, and were all 100% in PLS-DA model 2#, respectively, indicating excellent diagnostic accuracy (with all the three values in the range 0.9–1.0). In summary, the PLS-DA algorithm was effective in provenance classification of *B. balsamifera*.

**Figure 6.**
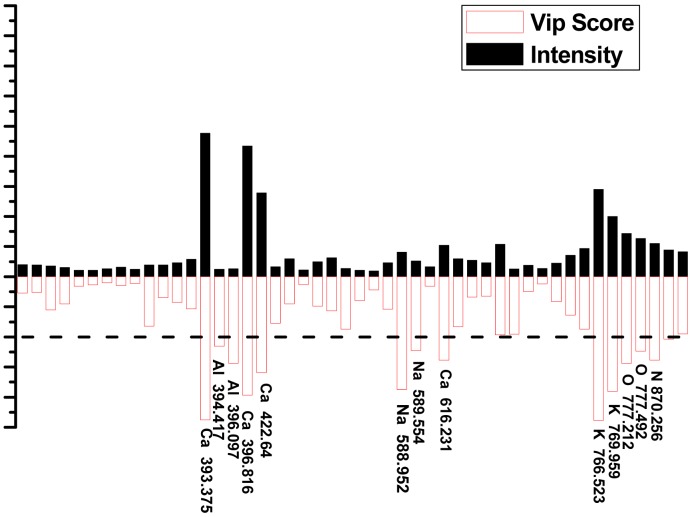
(Color online) The selected spectral lines and VIP scores of PLS-DA model 2#.

Since the goal is to separate one provenance from another, the data set can be down-selected *a priori*, in this case from complete spectra to selected spectral lines. The VIP scores of each class were used to determine how much each variable in the model contributed to the classification. In Figure 6, the selected spectral lines are compared to the VIP scores from PLS-DA model 2#. The selected spectral lines with higher VIP values are shown in Table 3. Higher VIP values showed greater importance among the classification results. VIP values between 0 and 1 were data that were not important in correct identification [45].

Elements such as Ca, K, Al, Na, and O played a significant role in provenance classification of *B. balsamifera* samples. The majority of the variables of complete spectra contributed significantly to the difference between classes. Some of the variables which did contribute to the model were due to the elemental composition of the samples suggesting that the natural environment affected the elemental composition of the plants.

## 4. Conclusions

In this study, multivariate analysis techniques were implemented to analyze LIBS spectra, focusing on discrimination of provenance of the herbal medicine *B. balsamifera*. All spectral data were pretreated by mean-centering prior to multivariate analysis.

PLS-DA was an effective method to discriminate the provenance of *B. balsamifera*. The two PLS-DA models using complete spectra and selected spectral lines as input variables provided a similar classification performance in this investigation. The down-selection of the dataset was specifically focused on the major elements (Ca, K, Al, Na and O) of *B. balsamifera*. Because the natural environment affects the growth and secondary metabolism of plants, the provenance of *B. balsamifera* can be classified via matrix elements.

Further research of LIBS technique for provenance study is underway. In order to further investigate the effects and obtain higher accuracy classification by LIBS technique, a large and diverse training dataset is needed. More measurement locations are required to obtain enough spectral information about composition variability. In addition, other multivariate analysis algorithms, such as support vector machines (SVM) or independent components analysis (ICA) [15] should be tested for their ability of provenance study. The results demonstrated the viability of LIBS for *B. balsamifera* provenance study. LIBS is a potential technique for future application in geoherbalism studies of medicinal herbs.

## Figures and Tables

**Table 1.** *Blumea balsamifera* DC samples from different geographical regions in China.

**Sample ID**	**Origins**	**Sample ID**	**Origins**
1∼4, 25∼31, 38∼40	Luodian, Guizhou	13∼18	Anlong, Guizhou
5∼7	Wuzhishan, Hainan	19∼21	Ceheng, Guizhou
8	Xingyi, Guizhou	22∼24	Wangmo, Guizhou
9∼12	Baisha, Hainnan	32∼37, 41∼95	Danzhou, Hainan

**Table 2.** Selected spectral lines and molecular bands of LIBS Spectra.

**Elements**	**Wavelength (nm)**	**Ref.**
C I	192.77; 247.725	[41]
Mg II	279.418; 280.123	[41]
Mg I	285.08; 383.825	[21]
Si I	288.031	[21]
Ca II	315.863; 317.92; 393.375	[41]
Ca II	370.627	[42]
Ca II	396.816	[43]
C-N	386.105; 387.08; 388.296	[41]
Al I	394.417; 396.097	[19]
Ca I	422.64; 558.842	[43]
Ca I	612.715; 616.231; 643.965	[41]
Ca I	646.214; 649.4; 714.856	[41]
Ca I	720.267	[41]
Sr II	407.789	[44]
Ca I	430.228; 442.64; 443.498	[41]
Ca I	445.441	[41]
Ba II	455.38; 493.388	[44]
C-C	516.672	[41]
Na I	588.952; 589.554	[19]
H I	656.315	[28]
Li I	670.754	[44]
N I	742.388; 746.918	[43]
N I	870.256; 871.046; 938.372	[43]
N I	744.306	[41]
K I	766.523; 769.959	[41]
O I	777.212; 777.492	[41]

**Table 3.** VIP scores for the selected spectral lines in PLS-DA calculations.

**Elements**	**VIPs**	**Elements**	**VIPs**
Ca II 393.375	2.377	Ca I 616.231	1.383
Al I 396.097	1.438	K I 766.523	2.386
Ca II 396.816	1.965	K I 769.959	1.905
Ca I 422.64	1.590	O I 777.212	1.439
Na I 588.952	1.872	N I 870.256	1.386
